# Development and Validation of a Deep Learning Model for Earlier Detection of Cognitive Decline From Clinical Notes in Electronic Health Records

**DOI:** 10.1001/jamanetworkopen.2021.35174

**Published:** 2021-11-18

**Authors:** Liqin Wang, John Laurentiev, Jie Yang, Ying-Chih Lo, Rebecca E. Amariglio, Deborah Blacker, Reisa A. Sperling, Gad A. Marshall, Li Zhou

**Affiliations:** 1 Department of Medicine, Brigham and Women's Hospital and Harvard Medical School, Boston, Massachusetts; 2 Department of Medicine, Brigham and Women's Hospital, Boston, Massachusetts; 3 School of Public Health, Zhejiang University School of Medicine, Hangzhou, Zhejiang, China; 4 Department of Neurology, Brigham and Women’s Hospital, Massachusetts General Hospital, Harvard Medical School, Boston, Massachusetts; 5 Department of Epidemiology, Harvard T. H. Chan School of Public Health and Department of Psychiatry, Massachusetts General Hospital, Harvard Medical School, Boston, Massachusetts

## Abstract

**Importance:**

Detecting cognitive decline earlier among older adults can facilitate enrollment in clinical trials and early interventions. Clinical notes in longitudinal electronic health records (EHRs) provide opportunities to detect cognitive decline earlier than it is noted in structured EHR fields as formal diagnoses.

**Objective:**

To develop and validate a deep learning model to detect evidence of cognitive decline from clinical notes in the EHR.

**Design, Setting, and Participants:**

Notes documented 4 years preceding the initial mild cognitive impairment (MCI) diagnosis were extracted from Mass General Brigham’s Enterprise Data Warehouse for patients aged 50 years or older and with initial MCI diagnosis during 2019. The study was conducted from March 1, 2020, to June 30, 2021. Sections of notes for cognitive decline were labeled manually and 2 reference data sets were created. Data set I contained a random sample of 4950 note sections filtered by a list of keywords related to cognitive functions and was used for model training and testing. Data set II contained 2000 randomly selected sections without keyword filtering for assessing whether the model performance was dependent on specific keywords.

**Main Outcomes and Measures:**

A deep learning model and 4 baseline models were developed and their performance was compared using the area under the receiver operating characteristic curve (AUROC) and area under the precision recall curve (AUPRC).

**Results:**

Data set I represented 1969 patients (1046 [53.1%] women; mean [SD] age, 76.0 [13.3] years). Data set II comprised 1161 patients (619 [53.3%] women; mean [SD] age, 76.5 [10.2] years). With some overlap of patients deleted, the unique population was 2166. Cognitive decline was noted in 1453 sections (29.4%) in data set I and 69 sections (3.45%) in data set II. Compared with the 4 baseline models, the deep learning model achieved the best performance in both data sets, with AUROC of 0.971 (95% CI, 0.967-0.976) and AUPRC of 0.933 (95% CI, 0.921-0.944) for data set I and AUROC of 0.997 (95% CI, 0.994-0.999) and AUPRC of 0.929 (95% CI, 0.870-0.969) for data set II.

**Conclusions and Relevance:**

In this diagnostic study, a deep learning model accurately detected cognitive decline from clinical notes preceding MCI diagnosis and had better performance than keyword-based search and other machine learning models. These results suggest that a deep learning model could be used for earlier detection of cognitive decline in the EHRs.

## Key Points

**Question:**

Can a deep learning algorithm applied to clinical notes detect evidence of cognitive decline before a mild cognitive impairment (MCI) diagnosis?

**Findings:**

In this diagnostic study, using clinical notes on 2166 patients preceding an MCI diagnosis, a deep learning algorithm was trained and validated for detecting cognitive decline using data sets with and without keyword filtering. The model trained in the data set with keyword filtering performed satisfactorily in the data sets without keyword filtering.

**Meaning:**

The results of this study suggest that a deep learning model can detect evidence of cognitive decline from notes preceding an MCI diagnosis, potentially facilitating earlier detection of cognitive decline in electronic health records.

## Teaser

This diagnostic study assesses the ability of a deep learning model to detect evidence of cognitive decline before a diagnosis of mild cognitive impairment using clinical notes from electronic health records.

## Introduction

There are nearly 6 million people diagnosed with Alzheimer disease (AD) at the stage of dementia in the US, and the prevalence increases dramatically with age.^1^ Mild cognitive impairment (MCI) and subjective cognitive decline (SCD) represent precursor stages that can serve as targets for early treatment.^2,3,4^ Early detection of cognitive decline can facilitate enrollment in clinical trials and early interventions.^5,6^ The US Food and Drug Administration recently approved aducanumab, a drug directed at the underlying pathologic characteristics of AD that clears amyloid plaques in the brain, to treat patients with AD.^7^ However, detecting patients with cognitive decline is challenging. There is an insufficient number of specialists with the necessary expertise (behavioral or cognitive neurologists, geriatric psychiatrists, geriatricians, and neuropsychologists) to see all at-risk patients. Instead, primary care physicians and other nondementia specialists have direct contact with these patients but not necessarily the time or tools needed for diagnosis.^8^

Systematically reviewing large electronic health record (EHR) data collected across patients’ full visit history within a health care system can facilitate early detection of cognitive decline by identifying when patients first reported signs or symptoms of cognitive decline to any health care professional. This documentation in turn may help trigger a more detailed evaluation in primary care settings and beyond and facilitate participant enrollment in clinical trials and early interventions. However, many obstacles exist to identifying patients with cognitive decline in the EHR. For early stages of cognitive decline, such as SCD, self-reported concerns about cognitive status do not imply a diagnosis of cognitive decline by a health care professional. Cognitive symptoms, concerns, and cognitive assessments may be merely documented in the health care professional’s notes, leaving such information difficult to identify and analyze. For later stages, when the decline becomes substantial enough to be measurable, patients may still have missed or delayed diagnosis of MCI or dementia.^8,9^

Current approaches to identifying cognitive decline from the EHR have limitations. These approaches commonly rely on billing codes or medications and are likely to be insensitive. Prior studies primarily focused on the stages of cognitive decline from MCI to dementia.^10,11,12^ Limited research has focused on detection of early cognitive decline preceding MCI or use of unstructured EHR data (clinical notes).

Clinical notes contain information that other EHR fields may not capture,^13^ and identifying evidence of cognitive decline from clinical notes can be complementary to evidence from structured EHR data. Detecting evidence of cognitive decline from clinical notes using manually curated keywords can be limited and lack accuracy, and manual medical record review is costly and nonscalable. In the present study, we aimed to develop and validate a deep learning model to automatically detect evidence of cognitive decline from clinical notes. Such an automated approach might be used to screen a large population of adults aged 50 years or older to identify early evidence of cognitive decline. We hypothesized that a deep learning algorithm can be trained with a relatively small set of manually labeled notes and applied to achieve this task.

## Methods

### Setting, Data Sources, and Study Sample

This study was conducted at Mass General Brigham (MGB, formerly Partners HealthCare), a large, integrated health care delivery system in Greater Boston, Massachusetts, from March 1, 2020, to June 30, 2021. We used data from MGB’s Enterprise Data Warehouse. We first identified patients aged 50 and older with an initial diagnosis of MCI (*International Statistical Classification of Diseases and Related Health Problems, Tenth Revision *code G31.84) in 2019. Then, we extracted patients’ clinical notes from the 4 years preceding the initial MCI diagnosis. Patients without any notes within the 4-year time window were excluded. The study was approved by the MGB Institutional Review Board with waiver of informed consent from study participants owing to secondary use of EHR data. In designing and reporting this study, we adhered to the Transparent Reporting of a Multivariable Prediction Model for Individual Prognosis or Diagnosis (TRIPOD) reporting guideline.^14^

### Definition of Cognitive Decline

Determining the presence of cognitive decline aimed to identify patients at any stage of cognitive decline, ranging from SCD to MCI to dementia. Therefore, cognitive decline can be captured by the mention of a cognitive concern, symptoms (eg, memory loss), diagnosis (eg, MCI, AD dementia), cognitive assessments (eg, Mini-Cog) (including patients with normal performance but with a note indicating a cognitive concern), or cognitive-related therapy or treatments (eg, cognitive-linguistic therapy). We focused on progressive cognitive decline that is likely to be consistent with or lead to MCI. Cases that were less likely progressive (eg, cognitive function has improved), transient (eg, temporarily forgetful or occasional memory loss due to medication intake [eg, codeine]), or reversible (eg, cognitive function affected soon after some event [eg, surgery, injury, or stroke]) were considered negative for cognitive decline. We also labeled sections of notes as negative when the record showed broader or uncertain indication of cognitive decline.

### Processing of Clinical Notes

Clinical notes can be long and contain many sections that are not relevant to cognitive assessments. Developing a machine learning model using notes with various lengths may require large training sets. Therefore, we first segmented clinical notes into sections. That is, our classification task was to identify whether a note section indicates that a patient has cognitive decline. We used the Medical Text Extraction, Reasoning and Mapping System natural language processing system^15^ to split clinical notes into sections, with each section assigned a normalized header.

### Development of a Labeled Data set

**Figure 1.**
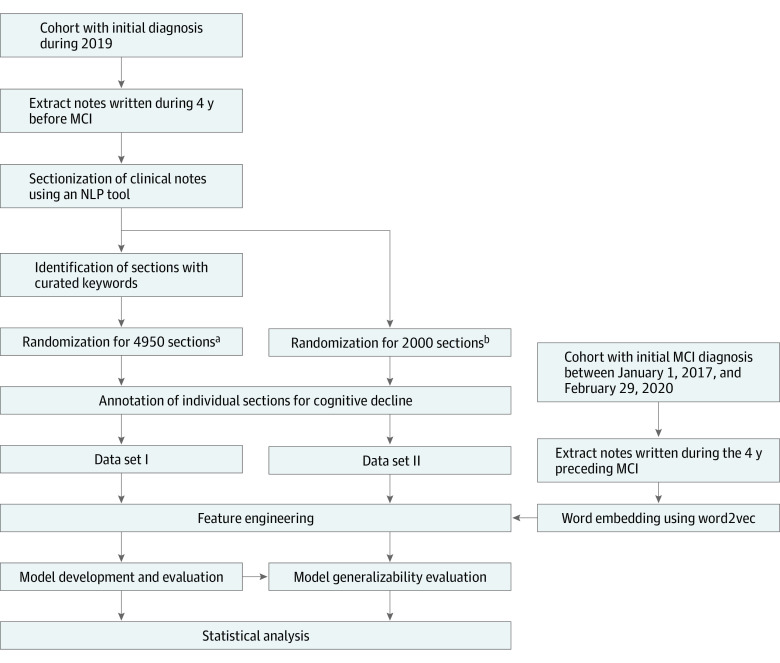
Flowchart of the Machine Learning Modeling for Cognitive Decline Detection Creation of 2 reference data sets for model development and evaluation and the creation of word embedding for the deep learning model. The study cohort was patients with initial mild cognitive impairment (MCI) diagnosis in 2019. Notes written during the 4 years preceding the MCI diagnosis were extracted. Two different randomization approaches were used for creating data sets I and II. The first randomization method. used for obtaining 4950 sections, considered an uneven distribution of sections among patients and section types. The second randomization method, used for obtaining 2000 sections, aimed to obtain a sample of sections from the entire corpus except the sections that were included in data set I. In addition, a larger corpus was used for training a word embedding model for the deep learning algorithm. NLP indicates natural language processing. ^a^Due to uneven distribution (for example, some patients might have more notes and some section types were more prevalent in the corpus) the randomization aimed to maximize the coverage of different patients and different section types. This randomization was performed among the sections with mention of keywords potentially related to cognitive decline. ^b^Randomization for sections of clinical notes from the entire corpus except the sections that were included in data set I.

We developed a labeled data set (data set I) to train, test, and compare multiple machine learning algorithms (Figure 1). To increase the positive case density of data set I, we applied a list of expert-curated keywords (Box) initially identified from cognitive-related topics based on topic modeling^16,17^ and enriched with experts’ knowledge a priori to filter for sections that likely contain indications of cognitive decline. Once we identified a list of sections that included the keywords, we randomized the sections by patient and section name to account for an uneven distribution of sections among patients and section types. Three annotators (ie, L.W., J.L., and Y.-C.L.) were trained with the assistance of subject matter experts (R.E.A. and G.A.M.) to label sections of clinical notes for cognitive decline. Annotators first individually labeled 150 sections and any conflicts were resolved by discussion with the subject matter experts. Then, in a second data set of 50 sections, the 3 annotators achieved good agreement with a Fleiss κ value of 0.83. An additional 4750 sections were each annotated by 1 of the annotators. Any cases for which labeling was uncertain were resolved by the subject matter experts. The final reference data set I contained 4950 sections.

Box. **Keywords Extracted From the Deep Learning Model vs Expert-Curated Keywords**

**Expert-Curated Keywords Developed Based on Topic Modeling Enriched With Knowledge A Priori**

Memory, delirium, dementia, psych^a^, neuro^a^, mental, alzheimer, confus^a^, mood, cognit^a^, forget^a^, agitat^a^, moca, montreal, mmse, remember, difficult, recall, function, word, evaluat^a^, score, drive, attention, mild, impairment, speech, question, disorientation, orientation, sleep, alter, exam, decline, worse, loss

**Keywords Extracted From the Deep Learning Model During Positive Prediction of Note Sections in Data Set I**

Memory, cognitive, dementia, impairment, neurocognitive, recall, decline, word, forgetful, cognition, MCI^b^, executive^b^, forgetfulness, alzheimer, remembering, MoCA, attention, deficits^b^, recalling, forgetting, finding^b^, words, visual^b^, forgets, naming^b^, difficulties, fluency^b^, delay^b^, alzheimers, retrieval^b^, visuospatial^b^, repetition^b^, remember, hearing^b^, cog^b^, trails^b^, language^b^, FTD^b^, frailty^b^, encoding^b^, developmental^b^, delayed^b^, behavioral^b^, amnestic^b^, phonemic^b^, MMSE, falls^b^, errors^b^, attentional, speech, span^b^, processing^b^, neurodegenerative, lapses^b^, HOH^b^, deficit^b^, correctly^b^, auditory^b^, years^b^, spatial^b^, solving^b^, semantic^b^, personality^b^, perseveration^b^, names^b^, multidomain^b^, moderately^b^, linguistic^b^, learning^b^, LBD^b^, items^b^, insight^b^, impaired^b^, immediate^b^, global^b^, functioning, functional, expressive^b^, died^b^, cube^b^, comprehension^b^, clock^b^, challenges^b^, category^b^, BNT^b^, aphasia^b^, amyloid^b^, age^b^, aforementioned^b^, abstraction^b^

Abbreviations: BNT, Boston Naming Test; FTD, frontotemporal dementia; HOH, hard of hearing; LBD, Lewy body disease; MCI, mild cognitive impairment; MMSE, Mini-Mental State Examination; MoCA, Montreal Cognitive Assessment.

^a^
Represent any characters.

^b^
Words were not included in the original keywords.

### Model Development and Validation

We implemented a hierarchical attention-based deep learning structure and 4 baseline machine learning algorithms, including logistic regression, random forest, support vector machine, and XGBoost.^18^ The deep learning algorithm was developed in a prior study^19^; it incorporates a convolutional neural network for the purpose of handling word variations, recurrent neural network for context, and attention layers for interpretation of the prediction. In the deep learning model, each note section was regarded as a sequence of tokens (including words and punctuation), with individual words represented by word embeddings, for which we used word2vec and trained 100-dimensional embeddings on a large corpus of 3 729 838 notes from 10 837 patients with an initial MCI diagnosis between January 1, 2017, and February 29, 2020.^20^ In the 4 baseline models, each section was represented with term frequency–inverse document frequency vectors based on n-grams (where n = 1).^21^ The algorithms were trained and tested through 5-fold cross-validation using data set I. The detailed parameters used for training the final deep learning model are specified in eTable 1 in the Supplement. We compared the deep learning model with the 4 baseline models in cognitive decline detection.

To interpret the prediction, we also identified words from the attention layer of the deep learning model that had high attention weight during the prediction. Word weights above the mean weights plus 2 SDs within individual sections were considered high attention.^19^ High attended words among the model-predicted positive cases were assembled and compared with the original curated keywords.

### Evaluation Without Keyword Filtering

Because data set I, used for model development, contained only sections with curated keywords, to assess the performance of the models on any sections regardless of keywords we created another labeled data set (data set II) to test the models trained on data set I. Data set II contained 2000 sections that were randomly selected from the entire note corpus, excluding the 4950 sections in data set I. We used this data set to evaluate model generalizability to note sections without keyword filtering.

### Statistical Analysis

We assessed the models using 2 performance metrics: the area under the receiver operating characteristic curve (AUROC) was used to evaluate the trade-off between sensitivity and specificity of the model across varying thresholds, and the area under the precision recall curve (AUPRC) provided complementary information for imbalanced data when the percentage of positive cases was low. Both AUROC and AUPRC were computed using the scikit-learn Python library (scikit-learn Developers). We estimated 95% CIs using 2000 bootstrap iterations (Python, version 3.7; Python Software Foundation).

## Results

We manually reviewed 4950 sections for data set I and 2000 sections for data set II (Table 1). Data set I represented 4745 notes and 1969 patients (1046 [53.1%] female; mean [SD] age, 76.0 [13.3] years). Data set II represented 1996 notes and 1161 patients (619 [53.3%] female; mean [SD] age, 76.5 [10.2] years). With some overlap of patients deleted, the unique population was 2166. The character-level length of the sections in data set I ranged between 6 and 9323 with mean (SD) length of 849 (936); for data set II, the character-level length ranged between 6 and 14 740 with mean of 463 (670). The positive predictive values (PPVs) of keyword search were low (ie, 0.294 in the data set I and 0.093 in data set II); with the application of keywords, the rate of positive cases increased from 3.45% (69 of 2000 sections) in data set II to 29.4% (1453 of 4950 sections) in data set I. In addition, in data set II, all the positive cases included at least 1 keyword.

**Table 1.** Characteristics for Data Sets I and II for the Development and Validation of Models for Identifying Cognitive Decline

Characteristic	Data set I	Data set II
Unique patients, No.	1969	1161
Sex, No. (%)		
Female	1046 (53.1)	619 (53.3)
Male	923 (46.9)	542 (46.7)
Age, mean (SD), y	76.0 (13.3)	76.5 (10.2)
Sections, No.	4950	2000
Notes, No.	4745	1996
Character-level length, mean (SD)	849 (936)	463 (669)
Keywords present, No. (%)	4950 (100)	740 (37.0)
Cognitive decline present in sections overall, No. (%)	1453 (29.4)	69 (3.5)
Cognitive decline present in sections with keywords (%)	1453 (29.4)	69 (9.3)

**Figure 2.**
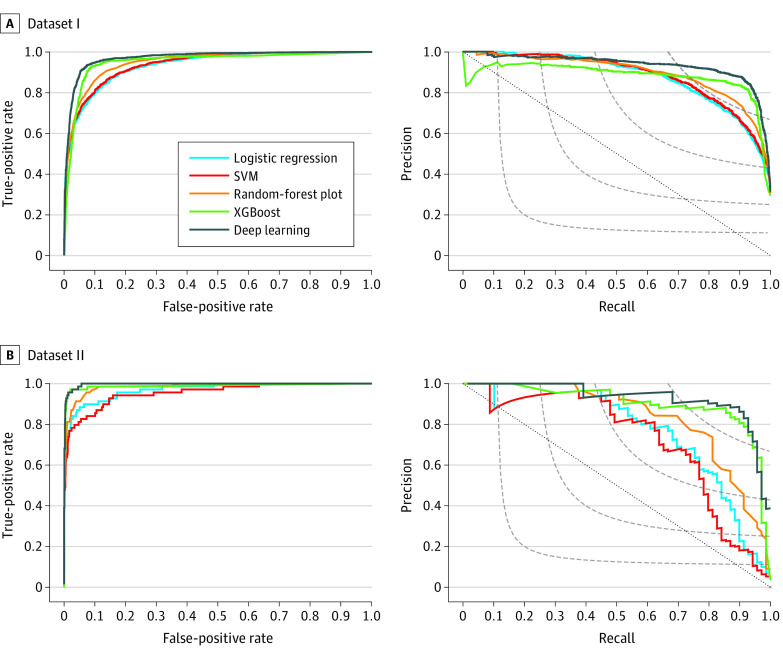
Machine Learning Model Performance Receiver operating characteristic curves and precision recall curves for the deep learning and baseline models (logistic regression, support vector machine [SVM], random forest, and XGBoost) in data sets I and II. A, Receiver operating characteristic curve (left panel) and precision recall curve (right panel) for data set I (4950 sections). B, Receiver operating characteristic curve (left panel) and precision recall curve (right panel) for data set II (2000 sections). In the left panels, gray-dashed curves contain all points in the precision/recall space whose F1 scores are the same, and the gray-dotted diagonal line indicates the performance based on random guessing.

Compared with the 4 machine learning algorithms (ie, random forest, logistic regression, support vector machine, and XGBoost), data set I of the deep learning model achieved the best performance, with an AUROC of 0.971 (95% CI, 0.967-0.976) and an AUPRC of 0.933 (95% CI, 0.921-0.944), representing an increase of 0.018 in AUROC and 0.051 in AUPRC compared with the best baseline model (ie, XGBoost). The data set I model was generalizable to data set II without applying keyword filtering, with an AUROC of 0.997 (95% CI, 0.994-0.999) and an AUPRC of 0.929 (95% CI, 0.870-0.969), representing an increase of 0.009 in AUROC and 0.031 in AUPRC over XGBoost. Table 2 and Figure 2 present the overall performance of the 5 models in terms of AUROC and AUPRC. With a 0.5 probability as the cutoff, the deep learning model could achieve a PPV of 0.848 and a sensitivity of 0.925 in data set I and a PPV of 0.771 and a sensitivity of 0.928 in data set II.

**Table 2.** Performance of 5 Machine Learning Models for Detecting Cognitive Decline From Clinical Notes

Model	AUROC (95% CI)	AUPRC (95% CI)
**Data set I (4950 sections)**		
Logistic regression	0.936 (0.929-0.943)	0.880 (0.867-0.893)
Random forest	0.950 (0.944-0.956)	0.889 (0.875-0.902)
SVM	0.939 (0.933-0.946)	0.883 (0.869-0.897)
XGBoost	0.953 (0.946-0.960)	0.882 (0.864-0.900)
Deep learning	0.971 (0.967-0.976)	0.933 (0.921-0.944)
**Data set II (2000 sections)**		
Logistic regression	0.969 (0.947-0.987)	0.762 (0.656-0.849)
Random forest	0.985 (0.972-0.994)	0.830 (0.746-0.898)
SVM	0.954 (0.924-0.979)	0.723 (0.618-0.822)
XGBoost	0.988 (0.969-0.998)	0.898 (0.830-0.957)
Deep learning	0.997 (0.994-0.999)	0.929 (0.870-0.969)

Abbreviations: AUPRC, area under the precision recall curve; AUROC, area under the receiver operating characteristic curve; SVM, support vector machine.

For individual sections, the weights of the words extracted from the attention layer during prediction revealed which words were predictive. eFigure 1 in the Supplement shows 3 example sections with positive and negative predictions and the words with high attention weights for the predictions.

By investigating the words of the positive cases in data set I that were assigned high attention weights in the deep learning model, we identified a list of keywords that were informative for positive prediction of cognitive decline (Box). Compared with the expert-curated keywords, the deep learning model had significantly expanded the keywords based on the labeled cases that we provided.

Although the deep learning model showed excellent performance, it made some incorrect predictions. We reviewed all false-positive and false-negative cases in data set I and summarized the reasons for the errors as well as examples (eTable 2 and eTable 3 in the Supplement).

## Discussion

In the present study, we aimed to develop a model to automatically detect evidence of cognitive decline from clinical notes and apply it to identify older adults with cognitive decline before any professional diagnosis of cognitive decline (ie, MCI, dementia). We developed 2 reference data sets; the first included a random sample of sections identified using keywords related to cognitive decline from notes documented before an initial MCI diagnosis, and the second included a random sample of sections without keyword filtering. We found that clinical notes before the initial MCI diagnosis contained evidence of cognitive decline. We developed and evaluated multiple machine learning models for detecting cognitive decline, among which the deep learning model achieved the best performance. Such a model can be a valuable tool for screening older patients to identify those with evidence of cognitive decline in clinical notes but not in structured EHR data (eg, problem lists, billing/diagnosis codes). Such patients could be in the early stages of cognitive decline or may have a delayed or missed diagnosis of MCI and/or dementia. Identifying these patients is important to facilitate widespread implementation of US Food and Drug Administration–approved early treatments and proper patient care. The model can also be valuable for identifying initial evidence of cognitive decline in the EHR to improve understanding of antecedent risk factors for early cognitive decline and predict long-term outcomes.

Most studies that detected cognitive decline used structured EHR data and focused on later stages of cognitive decline.^10,11,12,22^ Two studies used free-text notes for detecting MCI and/or dementia.^11,12^ The first used the Mayo Clinic Study of Aging population to assess keyword search for identifying patients with cognitive impairment and dementia among intensive care unit patients who received formal cognitive evaluation.^12^ The second study leveraged features from both structured and free-text EHR data in a logistic regression model to detect probable dementia in undiagnosed patients.^11^ To our knowledge, our study is the first to leverage deep learning algorithms and clinical notes for detecting evidence of cognitive decline before an MCI diagnosis.

The deep learning model was based on a study aiming to detect allergic reactions from free-text hospital safety reports.^19^ The algorithm was proven to be accurate and useful in allergic reaction detection. The present study noted the applicability of the deep learning algorithm to clinical notes. Because variable note length could affect model performance (eFigure 2 in the Supplement), we split the notes into sections to limit the computational demands of the model input while retaining sufficient content to allow the model to better understand the text. We found that this algorithm is feasible for classifying note sections for cognitive decline and believe it may be applicable to other conditions for which individual sections of notes can contribute to classification. However, situations that require more information or notes/reports that are ambiguous and difficult for even human labeling may pose challenges.

Because mentions of cognitive decline in clinical notes are sparse, we used a keyword search approach to increase the positive rate plus a randomization strategy to ensure broad coverage of patients and section types to create a more balanced data set. We found that this approach is efficient and feasible. Not only did the model reduce manual annotation effort, it also learned from the keyword-filtered cases, resulting in comparable performance when making predictions on the cases without keyword filtering.

Compared with a keyword-based search, the deep learning model can increase the PPV while maintaining comparable sensitivity. In data set II (Table 1), all the positive cases contained expert-curated keywords, which may indicate that keywords were sensitive in identifying cognitive decline from clinical notes. However, not all the cases with keywords were positive; in fact, the PPVs were low (ie, 0.294 in data set I and 0.093 in data set II). For the deep learning model, the PPVs increased (0.848 in data set I and 0.771 in data set II with 0.5 as probability cutoff) while maintaining high sensitivity (>0.920).

Compared with 4 baseline models, deep learning achieved significantly better AUROC and AUPRC for data set I. In data set II, owing to the imbalance of the data set, the AUROC scores of all 5 models had increased; however, all AUPRC scores had decreased except the XGBoost model scores. Despite a slight decrease in the AUPRC in data set II, the deep learning model still outperformed the XGBoost slightly. Although the improvement was small, the outcome could be greater when the model is applied to a large population (eg, >7 million adults aged 50 years or older in the MGB system). However, because the deep learning model requires advanced graphics processing unit machines, the XGBoost model can be an alternative when no graphics processing unit machine is available.

### Limitations

This study has several limitations. First, our models were trained using data from a single health care system and have not been validated using external data sets. Owing to variations in documentation among different health care systems, the word embeddings and models might need to be retrained to work well for data from other institutions. However, we believe that the overall approach and methods are replicable; our algorithm is publicly available in GitHub^19^ and we provided detailed parameters used for model training.

Second, although we used notes preceding initial MCI diagnosis, an MCI diagnosis in EHR records without any additional testing or confirmation is likely to result in a very mixed population, meaning that we may have included many patients without AD as the underlying cause, including several who will not progress to dementia, or even some who are already in a more advanced stage of cognitive decline (ie, dementia).^4^ Therefore, further efforts are warranted to classify those at earlier stages of cognitive decline due to AD.

Third, the model predicted some sections incorrectly. Weak evidence, uncommon way of expression, language variations or typographical errors (eg, conative for cognitive, impaiment for impairment, alzheimers for Alzheimer’s), or case sensitivity (our algorithm is case sensitive; for example, Moca, which appeared only once in data set I, was treated differently from MoCA and MOCA) led to false-negatives. To address these issues, we may automatically correct misspellings in clinical notes^23^ and turn off case sensitivity during model development. False-positive results were caused by a failure to recognize a negation of an indicator of cognitive decline, family history, or cognitive decline being mentioned as something to watch for (eg, in discussions of adverse effects or discharge instructions). However, the model also correctly predicted many such cases. We may use different approaches to address these issues, for example, rule-based natural language processing to handle negations and family history,^4^ increasing the size of the training data set, and/or implementing more advanced embedding techniques (eg, BERT; Google LLC).^24,25^ Our model is not meant to replace the clinician’s judgment and comprehensive clinical assessments by a dementia specialist, but to serve as a clinical decision-making tool to select patients at risk who would most benefit from such assessments, which are not widely available.

Fourth, the generalizability of the deep learning model to other stages of cognitive decline or a general population remains unknown, because it was developed and tested using a retrospective MCI cohort and implemented notes written during the 4 years preceding MCI diagnosis. Because we will use the model to screen prospective patients who do not yet have an MCI diagnosis, our next step will be to evaluate the performance of the deep learning model in detecting cognitive decline in a more general older population sample and beyond the 4-year time window.

Fifth, the model considered clinical notes in isolation from other EHR data, such as imaging data and cognitive scores. Integrating these data with our model poses several challenges. Few patients have imaging data and/or cognitive scores. In addition, radiology reports often lack the information (eg, the presence or absence of focal atrophy patterns) most helpful for predicting progressive cognitive decline. Another challenge with data integration involved cognitive testing, which was performed infrequently in nondementia specialist settings, and cognitive scores were often only documented as free text in notes. Any mention of cognitive tests in notes, even with normal scores, could indicate cognitive concerns prompting clinical evaluation. Such proxies for cognitive decline were classified as evidence of cognitive decline by the model. In follow-up studies, we will extract cognitive scores from the EHR and consider how to best leverage those data in models to predict future cognitive decline.

Sixth, our model may not distinguish well between reversible and progressive causes of cognitive decline. Although we have considered some transient or reversible contributors to cognitive decline, there are likely factors that were not considered. The models were designed to identify early cognitive symptoms 4 years before a diagnosis of MCI is made and so we had the advantage of knowing that the patients’ cognitive function ultimately decreased sufficiently to merit a diagnosis of MCI, which increases the likelihood of these early symptoms being relevant to progressive cognitive decline. However, once a patient receives a diagnosis of MCI, there is still a chance of reversion to normal cognition as opposed to progression to dementia. In addition, our model could not yet differentiate the stages of cognitive decline (eg, SCD, MCI, or dementia) or predict progression of early stages of cognitive decline to MCI or dementia. To better understand the cognitive decline identified by the model and long-term outcomes, future efforts are warranted to follow up a retrospective population with initial cognitive decline for outcomes using longitudinal EHR data.

## Conclusions

Early detection of cognitive decline offers promise for facilitating patient recruitment for clinical trials or identifying patients needing closer screening. A machine learning approach can be used to automatically identify evidence of cognitive decline from thousands or millions of clinical notes along patients’ longitudinal health records earlier than structured EHR fields, such as diagnosis codes, medications, and problem lists. The findings of this study suggest that a deep learning model that was trained on a relatively small set of labeled sections may be useful in detecting cognitive decline. In the future, we plan to apply the trained model to retrospectively identify patients’ first evidence of cognitive decline in the EHR, which will facilitate antecedent risk factor and outcome analyses.

## Notes

Supplement. **eTable 1.** Parameters Used in the Final Deep Learning Model **eTable 2.** Error Analysis for Potential Factors Leading to False Positives in the Deep Learning Model **eTable 3.** Error Analysis for Potential Factors Leading to False Negatives in the Deep Learning Model **eFigure 1.** Example Sections with Attention Heat Maps **eFigure 2.** Model Performance by Section Length
