# Supplementary material for: Development and Validation of a Deep Learning Model for Earlier Detection of Cognitive Decline From Clinical Notes in Electronic Health Records
Source: JAMA Netw Open. 2021 Nov 18;4(11):e2135174. doi: 10.1001/jamanetworkopen.2021.35174 (PMC8603078; doi:10.1001/jamanetworkopen.2021.35174)
Supplement: Supplement. — eTable 1. Parameters Used in the Final Deep Learning Model eTable 2. Error Analysis for Potential Factors Leading to False Positives in the Deep Learning Model eTable 3. Error Analysis for Potential Factors Leading to False Negatives in the Deep Learning Model eFigure 1. Example Sections with Attention Heat Maps eFigure 2. Model Performance by Section Length [file jamanetwopen-e2135174-s001.pdf]

Supplementary Online Content

Wang L, Laurentiev J, Yang J, et al. Development and validation of a deep learning model for earlier detection of cognitive decline from clinical notes in electronic health records. *JAMA Netw Open*. 2021;4(11):e2135174. doi:10.1001/jamanetworkopen.2021.35174

eTable 1. Parameters Used in the Final Deep Learning Model

eTable 2. Error Analysis for Potential Factors Leading to False Positives in the Deep Learning Model

eTable 3. Error Analysis for Potential Factors Leading to False Negatives in the Deep Learning Model

eFigure 1. Example Sections with Attention Heat Maps

eFigure 2. Model Performance by Section Length

This supplementary material has been provided by the authors to give readers additional information about their work.

© 2021 Wang L et al. *JAMA Network Open.*

eTable 1. Parameters Used in the Final Deep Learning Model

**Parameter**	**Value**
Word embedding dimensions	100
Character embedding dimensions	30
Min word frequency cut-off	5
LSTM hidden layers	1
LSTM hidden layer size	200
CNN hidden layers	4
CNN hidden layer size	50
Optimizer	SGD
Batch size	10
Learning rate	0.05
Epochs (iterations)	30

Abbreviations: LSTM, long short-term memory; CNN, convolutional neural network; SGD, stochastic gradient descent.
Note: the parameters were used for training the final deep learning model. The deep learning algorithm is available in GitHub: https://github.com/jiesutd/AllergicEvent

© 2021 Wang L et al. *JAMA Network Open.*

eTable 2. Error Analysis for Potential Factors Leading to False Positives in the Deep Learning Model

**Potential Factors Contributing to False Predictions**	**Examples of False Positives**
Cognitive decline attributed to people other than patients (e.g., family members, friends)	Discharge plan/ / involved: now has 24/7 care from freedom healthcare, glss is involved, per hcp who lives out of State, plan is to reassess needs on Monday and make necessary adjustment, pt's husband lives at the house and has dementia, noted to be pacing through this assess ment, pt agrees to sn and hha (says only does sponge bath) services, PT/OT eval.
Negation	Demographics Resides in assisted living or nursing home?-no. Older than 75?-no. Handicap or wheelchair bound?-no. Cognitive disability or unable to give consent?-no. Does patient have any of following conditions?. Heart failure-no. Systemic pulmonary shunt-no. MI/stroke within 12 months-no. Angioplasty-no. Dysrhythmia-no. Diabetes-no. Heart valve abnormalities-no.
Improvement of memory	HPI Patient states she is feeling better, thinks her memory improved and she also got her drivers license back. She had a repeat evaluation of the AAA, and the ultrasound showed ectasia, not aneurysm. We can recheck in 1-2 yrs, or prn symptoms.
Instructions	Activity While in Hospital: Ambulation ad lib with S for hemodynamic monitoring. Positioning: OOB to chair as much as tolerated. Other Consults: Occupational Therapy if concern for cognition. Medical Update: Approaching medical readiness for d/c, LE compression stockings and abdominal binder not donned for session.
General Knowledge/Rational	In case family asks the rational for NOT just checking urines all the time-we do not do as over treatment causes med side effects and resistance making future real UTIs harder to treat. Establishing the diagnosis in older patients is complicated due to the higher prevalence of chronic urinary symptoms and cognitive impairment, which make it difficult to identify specific UTI symptoms. This diagnostic challenge, in addition to the high prevalence of asymptomatic bacteriuria in this population, often leads to overdiagnosis and unnecessary treatment. Therefore, it is important to have a high threshold for urine testing to avoid this pitfall. We recommend urine testing only in the presence of classic signs and symptoms of UTI (acute dysuria, new or worsening urgency or frequency, new incontinence, gross hematuria, and suprapubic or costovertebral angle tenderness) or physiologic signs of serious acute illness (eg, fever, other major vital sign abnormalities, changes in level of consciousness). Signs of systemic infection, particularly in the absence of urinary symptoms, should prompt evaluation for other sources of infection.

Note: The text of the cases above reflected what were stored in the database, except that the names, dates, and locations were de-identified. The grey text was manually marked, showing no evidence of cognitive decline, but which may have led to false predictions.

© 2021 Wang L et al. *JAMA Network Open.*

eTable 3. Error Analysis for Potential Factors Leading to False Negatives in the Deep Learning Model

**Potential Factors Contributing to False Predictions**	**Examples of False Negatives**
Weak evidence	Counseling and coordination of care 05/21/2016. Abdominal aortic aneurysm 05/21/2016. Bradycardia 06/27/2016. Pulmonary nodule 06/27/2016. Coronary artery disease 06/27/2016. Abscess 10/14/2018. Comedonal acne 10/28/2018. Ischemic cardiomyopathy 12/28/2018. Forgetfulness 10/28/2018. Melanoma in situ of face 10/28/2018. Actinic keratosis 10/28/2018.
	Discharge to SNF date: 12/7/2019. DC to home/ALF: 01/22/2020. Now residing at ALF/Memory Unit @ PLACE. Fell at home, fractured hip. Surgically repaired 12/4/19. Also, present is the patient's personal care attendant..
Typo or language variations	REASON FOR REFERRAL Pt is being referred for a consultation regarding mild conative disorder. Pt is scheduled to see Dr.Smith on 11/25/20. CLINICALLY URGENT: N. IF YES, WHY:. SUICIDAL THOUGHTS: N. THOUGHTS OF HARMING OTHERS: N. SUBSTANCE USE: N. IF "YES", PLEASE SPECIFY TYPE, FREQUENCY, CURRENT/PAST SUBSTANCE ABUSE TREATMENT. PATIENT BOOKED FOR AN EVALUATION: Y. EMAILED PCP/REFERRING MD (IF NOT PCP):??: Y.
	Family History Family History Problem Relation Age of Onset. Arthritis Mother. Diabetes Father. Arthritis Brother. Esophageal cancer Neg Hx. Stomach cancer Neg Hx. Colon cancer Neg Hx. Geriatric ROS:. Emotional:reports mood has improved, she feels more active, has been going out more. Health Attitude: good. Motivation: usual. LH/Dizziness: occasionally; getting better with exercises which she does in AM and before bed. Vision: corrected; reading glasses. Hearing: within normal limits. Dentures: none. Balance: impaired. Falls last 6 months: no. Memory: memory impaiment that does not interfere with daily function. GI: continent. GU: continent.
	PMH includes HTN, hyperlipidemia, PSVT s/p cardioversion 2017, exertional substernal chest discomfort usually resolves with rest but occasional nitroglycerin required (stress test normal 9/2018), OSA - uses CPAP, BPH, fatty liver, NIDDM, A1c 6.2 (7/2019),Am glucose runs 106, polyneuropathy,neck pain, OA, depression, anxiety and mild alzheimers - on med. Pt. Coming in for EGD/Colonoscopy. Informed Consent:. Anesthetic plan and risks discussed with: patient. Final anesthesia plan to be determined on the day of surgery by the primary anesthesia team.
Negations	Subjective memory/cognitive complaints unchanged. Headaches "off and on". Did not try reduced alprazolam dose. PFSH updates today:. Describes h/o extensive DV exposure in previous relationship. Recalls very brief trial of prazosin; can't say whether it helped.
Transcription error or uncommon way of expression	Exercise Low Intensity 3-4 days a week. ADL:. No issues with: dressing, bathing, walking, shopping, housekeeping and financial management. Services: none. Advance Care Planning:. Health Care Agents. Jacobson, Beverly .Primary Health Care Agent (Proxy form on file) - Spouse Not Active. No MOLST on file. Advance Care/End of Life Planning Discussed: Wife is healthcare proxy. Depression Screening:. Depression Screening 11/12/2019. PHQ-2 Score 0. Cognition:. The patient has had neuropsychologic testing and see neurologist and he does have some problem with "and coding intake" as well as storage of new information and he was recommended to carry note and keep track of his daily actions on computer. Hearing/Vision:. Hearing ROS: hearing is okay. Sees eye MD. STRIDE Falls Risk:. Falls Risk 11/12/2019. Have you fallen and hurt yourself in the past year? No. Have you fallen 2 or more times in the past year? No. Are you afraid that you might fall

Potential Factors Contributing to False Predictions	Examples of False Negatives
	because of balance or walking problems? No. Home Safety. Who checks on patient: Spouse. Frequency: daily. The following home safety topics were reviewed:. loose rugs. Patient is not taking any prescription opioid medication(s).. 5-10 year preventative screening recommendations discussed.

Note: The text of the cases above reflected what were stored in the database, except that the names, dates, and locations were de-identified. The grey text was manually marked, showing the evidence of cognitive decline, but which may have not been detected by the deep learning model.

© 2021 Wang L et al. *JAMA Network Open.*

eFigure 1. Example Sections with Attention Heat Maps. The attention heat maps show words predictive for cognitive decline in example positive and negative predicted cases

A. Attention to words contributing to the prediction of positive cases

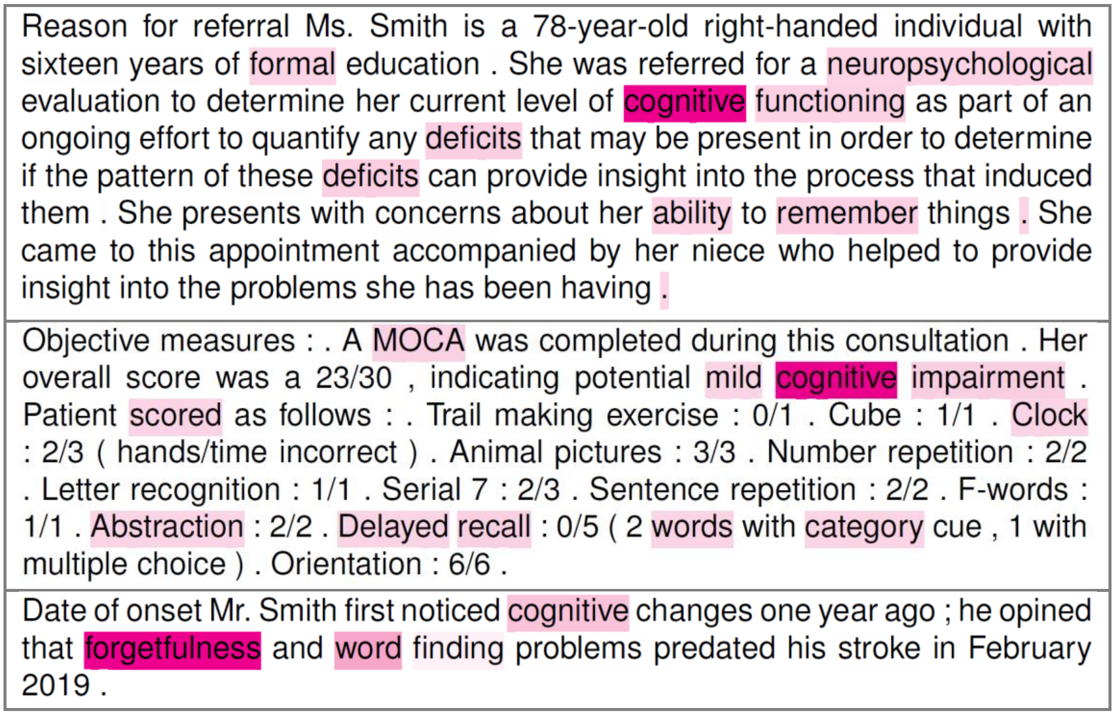
Reason for referral Ms. Smith is a 78-year-old right-handed individual with sixteen years of formal education . She was referred for a neuropsychological evaluation to determine her current level of cognitive functioning as part of an ongoing effort to quantify any deficits that may be present in order to determine if the pattern of these deficits can provide insight into the process that induced them . She presents with concerns about her ability to remember things . She came to this appointment accompanied by her niece who helped to provide insight into the problems she has been having .

Objective measures : . A MOCA was completed during this consultation . Her overall score was a 23/30 , indicating potential mild cognitive impairment . Patient scored as follows : . Trail making exercise : 0/1 . Cube : 1/1 . Clock : 2/3 (hands/time incorrect) . Animal pictures : 3/3 . Number repetition : 2/2 . Letter recognition : 1/1 . Serial 7 : 2/3 . Sentence repetition : 2/2 . F-words : 1/1 . Abstraction : 2/2 . Delayed recall : 0/5 (2 words with category cue , 1 with multiple choice) . Orientation : 6/6 .

Date of onset Mr. Smith first noticed cognitive changes one year ago ; he opined that forgetfulness and word finding problems predated his stroke in February 2019 .

B. Attention to words contributing to negative cases

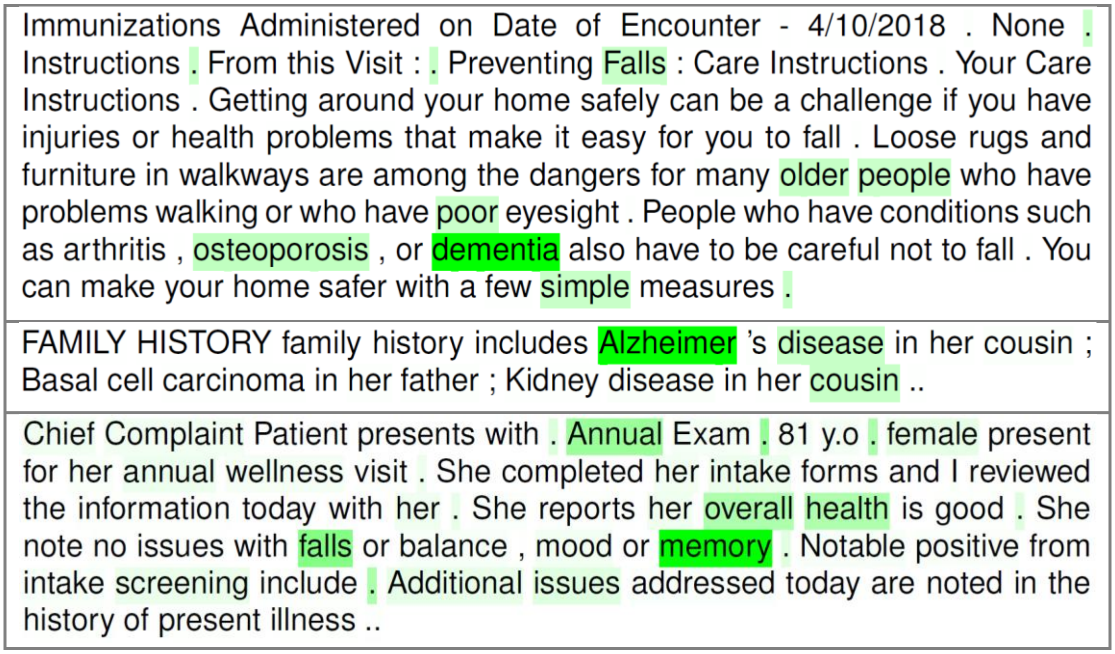
Immunizations Administered on Date of Encounter - 4/10/2018 . None . Instructions . From this Visit : . Preventing Falls : Care Instructions . Your Care Instructions . Getting around your home safely can be a challenge if you have injuries or health problems that make it easy for you to fall . Loose rugs and furniture in walkways are among the dangers for many older people who have problems walking or who have poor eyesight . People who have conditions such as arthritis , osteoporosis , or dementia also have to be careful not to fall . You can make your home safer with a few simple measures .

FAMILY HISTORY family history includes Alzheimer 's disease in her cousin ; Basal cell carcinoma in her father ; Kidney disease in her cousin ..

Chief Complaint Patient presents with . Annual Exam . 81 y.o . female present for her annual wellness visit . She completed her intake forms and I reviewed the information today with her . She reports her overall health is good . She note no issues with falls or balance , mood or memory . Notable positive from intake screening include . Additional issues addressed today are noted in the history of present illness ..

Note: Names, dates, and locations were de-identified in the example cases.

© 2021 Wang L et al. *JAMA Network Open.*

eFigure 2. Model Performance by Section Length

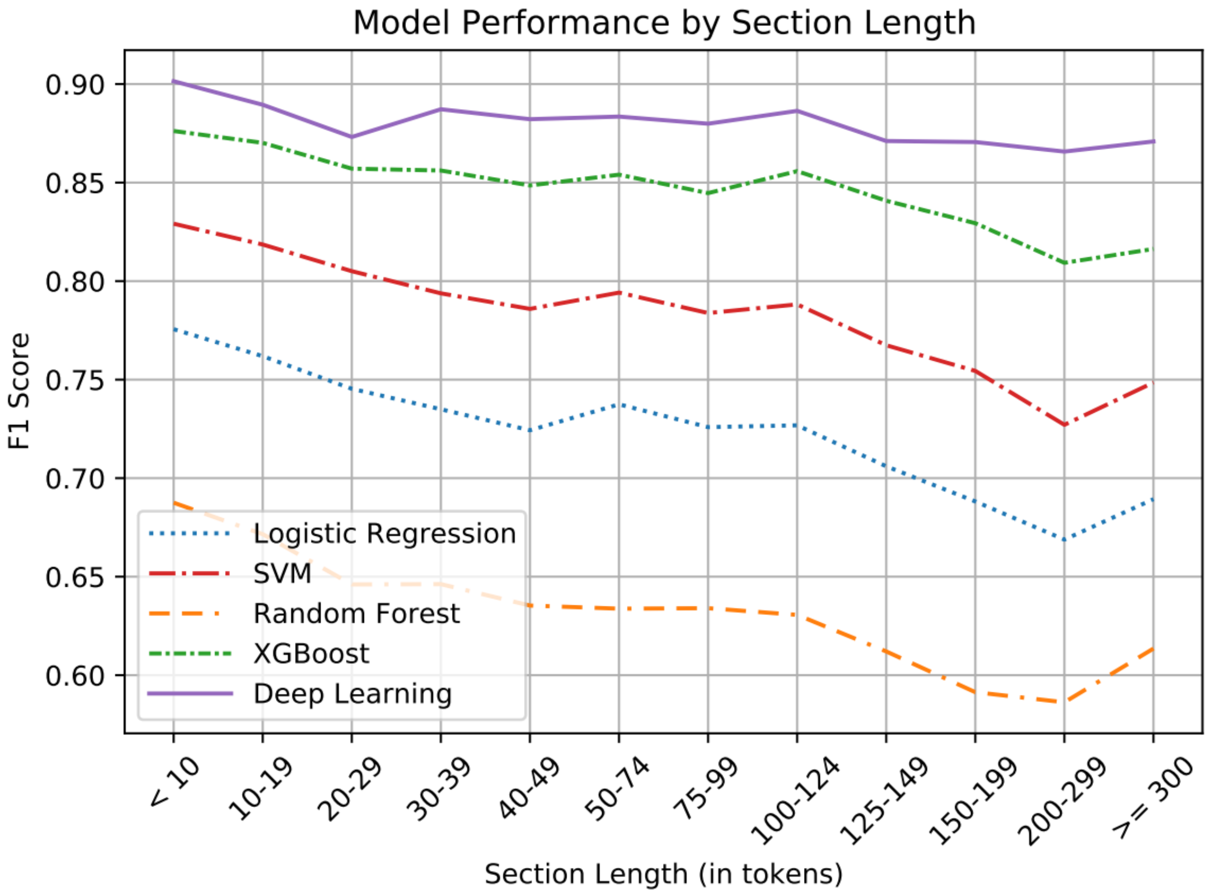

© 2021 Wang L et al. *JAMA Network Open.*
